# Oncologic Safety of Facial‐Vessel–Based Buccinator Myomucosal Flaps for Tongue and Oral Floor Reconstruction: A Retrospective Multicenter Case–Control Study

**DOI:** 10.1002/hed.70082

**Published:** 2025-10-23

**Authors:** Luigi Angelo Vaira, Olindo Massarelli, Jerry Polesel, Andrea Ferri, Carlos M. Chiesa‐Estomba, Ignacio Navarro Cuéllar, Giuseppe Consorti, Giulio Cirignaco, Marco Cucurullo, Giovanni Salzano, Sourabh Padmanabhan, Abhijith George, Marzia Petrocelli, Javeria Ali, Jose A. González‐Garcia, Alfonso Manfuso, Tareck Ayad, Carlos Navarro Cuéllar, Fabiola Giudici, Jerome R. Lechien, Shawn T. Joseph, Chiara Copelli, Federico Biglioli, Silvano Ferrari, Giacomo De Riu

**Affiliations:** ^1^ Maxillofacial Surgery Operative Unit, Department of Medicine, Surgery and Pharmacy University of Sassari Sassari Italy; ^2^ Maxillofacial Surgery Operative Unit, Department of Medical Biotechnology University Hospital of Siena Siena Italy; ^3^ Unit of Cancer Epidemiology Centro di Riferimento Oncologico di Aviano (CRO) IRCCS Aviano Italy; ^4^ Maxillo‐Facial Surgery Unit, Head Neck Department University Hospital of Parma Parma Italy; ^5^ Department of Otorhinolaryngology, Biodonostia Research Institute, Osakidetza Donostia University Hospital San Sebastian Spain; ^6^ Maxillofacial Surgery Department Hospital Gregorio Marañon, Universidad Complutense de Madrid Madrid Spain; ^7^ Division of Maxillofacial Surgery, Department of Neurological Sciences Marche University Hospitals—Umberto I Ancona Italy; ^8^ Department of Biomedical Sciences and Public Health Polytechnic University of Marche Ancona Italy; ^9^ Maxillofacial Surgery Department, San Paolo Hospital, ASST Santi Paolo e Carlo University of Milan Milan Italy; ^10^ Maxillofacial Surgery Unit, Department of Neurosciences, Reproductive and Odontostomatological Sciences University Federico II Naples Italy; ^11^ Institute of Head and Neck Sciences, Aster Medcity Kochi India; ^12^ VPS Lakeshore Hospital Kochi India; ^13^ Maxillofacial Surgery Operative Unit; Bellaria and Maggiore Hospital AUSL Bologna Bologna Italy; ^14^ Maxillofacial Surgery Unit, Interdisciplinary Department of Medicine University of Bari “Aldo Moro” Bari Italy; ^15^ Division of Otolaryngology‐Head and Neck Surgery Centre Hospitalier de l'Université de Montréal Montreal Canada; ^16^ Division of Laryngology and Broncho‐Esophagology, Department of Otolaryngology and Head and Neck Surgery, EpiCURA Hospital, UMONS Research Institute for Health Sciences and Technology University of Mons Mons Belgium; ^17^ Head and Neck Surgery Unit Elsan Polyclinic of Poitiers Poitiers France

**Keywords:** buccinator myomucosal flap, facial artery musculomucosal flap, oncologic safety, oral floor reconstruction, squamous cell carcinoma, tongue reconstruction

## Abstract

**Background:**

Buccinator myomucosal flaps (BMFs) have been proposed as a reconstructive solution for defects of the tongue and oral floor; however, their harvest requires preservation of the facial artery and vein. This study aimed to evaluate the oncologic safety of this approach compared with free fasciocutaneous flaps (FFF).

**Methods:**

A retrospective multicenter case–control study including cT1–T3 cN0 tongue/oral floor squamous cell carcinoma was performed. Cases received BMFs while controls received FFFs. The primary endpoint was progression‐free survival (PFS) tested for non‐inferiority. Secondary endpoints were overall survival (OS), disease‐specific survival (DSS), and cumulative incidence of local, regional, and distant recurrence using competing‐risk methods.

**Results:**

A total of 615 patients (BMF *n* = 390; FFF *n* = 225) with comparable baselines were included. Five‐year PFS was 69.8% (BMF) versus 66.2% (FFF); adjusted HR (FFF vs. BMF) 0.87 (95% CI: 0.43–1.78), meeting non‐inferiority. Five‐year OS was 77.9% versus 73.5%. Cumulative incidence of recurrence was similar: local 7.5% versus 8.3%, regional 6.3% versus 6.1%, and distant 2.1% versus 1.7%.

**Conclusions:**

Preservation of the facial artery and vein during selective neck dissection did not compromise oncologic outcomes. Facial‐vessel–based BMFs are a valid option for small‐to‐medium tongue/oral floor defects in appropriately selected cN0 patients when meticulous level I clearance is performed and a pull‐through resection is not required.

## 1 Introduction

Resection of squamous cell carcinoma (SCC) of the tongue and oral floor frequently results in mucosal defects that, even when limited in size, may significantly impair speech, swallowing, and overall quality of life [1, 2, 3]. Reconstruction of these defects remains challenging, as no currently available technique provides a true “like‐with‐like” replacement of the lost mucosa [4]. Free fasciocutaneous flaps, particularly the radial forearm free flap (RFFF) [5] and the anterolateral thigh (ALT) flap [6], are currently considered the reconstructive standard. However, these flaps are associated with donor site morbidity, require a dual surgical team, and replace mucosa with skin, a tissue that lacks sensitivity recovery and is prone to scar contracture during healing [7, 8].

Buccinator myomucosal flaps (BMFs) have been proposed as an alternative for small to medium‐sized oral cavity defects [9, 10, 11, 12, 13]. These flaps provide tissue similar in texture and sensibility to the resected mucosa, thereby favoring sensory recovery and functional restoration of phonation and swallowing [14, 15, 16, 17]. Their use may also reduce the need for free flaps in appropriately selected patients, simplifying reconstructive management.

Despite these advantages, concerns persist regarding their oncologic safety. Harvesting a BMF requires preservation of the facial artery and vein during lateral neck dissection, which may compromise surgical radicality in patients staged clinically as N0 but subsequently upstaged after histopathologic examination [18, 19]. Furthermore, some authors have speculated that replacing oral mucosa with tissue exposed to the same carcinogenic field could predispose one to the development of metachronous malignancies within the flap [20, 21].

The oncologic safety of preserving facial vessels has been extensively studied for other regional flaps, such as the submental flap [22, 23, 24], with most reports showing no increase in recurrence risk when meticulous perivascular node dissection is performed. In contrast, evidence on BMFs remains scarce. To date, only one small retrospective series has specifically addressed this issue. Ferrari et al. [25] analyzed 50 patients reconstructed with facial artery myomucosal flaps and reported that preservation of the facial vessels did not increase the rate of regional recurrences. However, that study was limited by its small sample size, heterogeneity of tumor sites and T‐stages, and the lack of a control group reconstructed with free flaps, in which facial vessels were therefore sacrificed.

To overcome these limitations, a large multicenter retrospective case–control study was conducted to evaluate the oncologic safety of BMFs compared with free fasciocutaneous flaps. The primary objective was to assess whether preservation of the facial artery and vein during lateral neck dissection compromises local or regional control in patients with clinically N0 SCC of the tongue and oral floor.

## 2 Materials and Methods

This is a retrospective, multicenter, non‐inferiority case–control study of patients with SCC of the tongue and/or oral floor clinically staged cT1–T3 cN0 who underwent tumor resection with elective neck dissection and immediate reconstruction. The exposure group received BMF reconstruction based on the facial artery and vein, whereas controls received FFF with the sacrifice of the facial vessels during neck dissection.

The study involved eight centers in Europe and one in India: the Maxillofacial Surgery Unit of the University Hospital of Sassari (Italy); the Maxillofacial Surgery Unit of the University Hospital of Parma (Italy); the Maxillofacial Surgery Unit of the San Paolo Hospital of Milan (Italy); the Maxillofacial Surgery Unit of the Bellaria–Maggiore Hospital of Bologna (Italy); the Maxillofacial Surgery Unit of the University Hospital of Bari (Italy); the Division of Maxillofacial Surgery of the University Hospital of Ancona (Italy); the Department of Otorhinolaryngology of the Donostia University Hospital of San Sebastián (Spain); the Department of Oral and Maxillofacial Surgery of the Hospital General Universitario Gregorio Marañón of Madrid (Spain); and the Department of Head and Neck Surgical Oncology of the Lakeshore Hospital of Kochi (India).

The study was conducted in accordance with the ethical standards of the institutional and/or national research committee and with the 1964 Declaration of Helsinki and its later amendments. The protocol was approved by the Regional Ethics Committee of Sardinia (approval No. 04, January 21, 2025). This report adheres to the STROBE (Strengthening the Reporting of Observational Studies in Epidemiology) statement for case–control studies.

Inclusion criteria were: (1) primary SCC of the tongue or oral floor; (2) clinical–radiologic stage cT1–T3 cN0; (3) elective selective neck dissection; (4) immediate reconstruction with either BMF (facial vessels preserved) or FFF (facial vessels sacrificed); and (5) minimum follow‐up of 24 months or death within 24 months. Exclusion criteria were prior head and neck malignancy, cT4 tumors, cN+ neck, positive (R+) margins, or incomplete documentation.

Primary tumor resection followed institutional standards. Elective neck dissection typically encompassed levels I–III or I–IV. In the BMF cohort, the facial artery and vein were carefully preserved, and perivascular lymphatic tissue was dissected en bloc with level I nodes; flap type [26] was selected according to defect site and surgeon preference. In the FFF cohort, facial vessels were included in the neck specimen and thus sacrificed.

Adjuvant radiotherapy or chemotherapy was administered according to final pathology and multidisciplinary tumor board recommendations. Patients were followed per center protocols; dates and sites of recurrence and vital status were recorded prospectively in each institution's medical record and extracted into a centralized database.

The primary endpoint was progression‐free survival (PFS), defined as the time from surgery to the first event (local, regional, or distant recurrence) or death from any cause, whichever occurred first. Secondary endpoints were overall survival (OS), disease‐specific survival (DSS), and cumulative incidence of local, regional, and distant recurrence.

Eligible cases were identified consecutively at each site. Two trained researchers per center abstracted data into a standardized matrix; datasets were anonymized, centralized at the coordinating center, and cross‐checked for internal consistency before statistical analysis. For each included patient, age at surgery, sex, and date of surgery were recorded; index tumor characteristics were documented, including primary site, clinical and pathological TNM stage, histologic grade, number and neck level(s) of metastatic lymph nodes, and extranodal extension; operative details were collected, including the side and extent of selective neck dissection, whether the facial artery and vein were sacrificed, and the reconstructive technique employed; adjuvant therapies were noted; and, at follow‐up, vital status, date of last contact, and—when applicable—the site (local, regional, or distant) and date of first recurrence were documented.

Descriptive statistics summarized demographic, tumor, and treatment characteristics: medians and interquartile range (IQR) for quantitative variables and proportions for qualitative variables. Between‐group comparisons used Mann–Whitney *U* for quantitative variables and *χ* ^2^ test for qualitative variables. Statistical significance was claimed for *p* < 0.05.

Time‐to‐event analyses for PFS (primary outcome) used Kaplan–Meier estimates compared by log‐rank test [27]. For each patient, the time at risk was calculated from the date of surgery to the date of local recurrence, regional recurrence, distant recurrence, death, or last follow‐up, whichever occurred first. Analyses were truncated at 5 years. Cox proportional hazards models estimated hazard ratios (HRs) with 95% confidence intervals (CIs), with multivariable regression to account for confounders (i.e., gender, age, cancer site, pathological T stage, pathological N stage upgrade, grading, extranodal extension, and adjuvant treatment). Non‐inferiority of BMF versus FFF was confirmed if the lower bound of the 95% CI for the HR < 1. Secondary outcomes (OS, DSS) were analyzed with the same framework.

Based on the primary objective, 379 patients per group were required to evaluate non‐inferiority (margin HR = 1.20, *α* = 0.05, *β* = 0.20; 80% power). Given the retrospective design, no losses to follow‐up were anticipated.

To account for competing risk due to death, local, regional, and distant recurrences were evaluated using cumulative incidence, and differences according to strata were tested using Gray's test [28]. Furthermore, HRs were adjusted for competing risk according to the Fine–Gray model. Statistical analyses were conducted with SAS 9.4.

## 3 Results

A total of 615 patients met the inclusion criteria: 390 underwent reconstruction with a facial‐vessel–based BMF (BMF group) and 225 with a FFF (FFF group); the mean follow‐up duration was 56.2 ± 29.9 months (range 1–126 months). The two groups (Table 1) were homogeneous for age (median 64 years in BMF vs. 62 years in FFF; *p* = 0.233), sex (female 42.6% in BMF vs. 40.9% in FFF; *p* = 0.685), primary site (*p* = 0.474), clinical T stage (*p* = 0.790), histologic grade (*p* = 0.951), pathological T stage (*p* = 0.716), and pathological N stage (*p* = 0.613). Upstaging from cN0 to pathologic N+ occurred in 20.2% overall (20.3% BMF vs. 20.0% FFF; *p* = 0.939). Extranodal extension was positive in 2.6% of patients in the BMF group and in 2.1% in the FFF group (*p* = 0.462).

**TABLE 1:** Distribution of patients according to clinical characteristics.

	All (*n* = 615)		Cases (*n* = 390)		Controls (*n* = 225)		*χ* ^2^ test
	*N*	(%)	*N*	(%)	*N*	(%)	
Age at surgery (years)							
Median (IQR)	62	(51–70)	64	(51–71)	62	(50–68)	*p* = 0.233^a^
Gender							
Female	258	(42.0)	166	(42.6)	92	(40.9)	*p* = 0.685
Male	357	(58.0)	224	(57.4)	133	(59.1)	
Cancer site							
Tongue	361	(58.7)	232	(59.5)	129	(57.3)	*p* = 0.474
Oral floor	177	(28.8)	114	(29.2)	63	(28.0)	
Tongue and oral floor	77	(12.5)	44	(11.3)	33	(14.7)	
Clinical T stage							
cT1	143	(23.3)	94	(24.1)	49	(21.8)	*p* = 0.790
cT2	422	(68.6)	264	(67.7)	158	(70.2)	
cT3	50	(8.1)	32	(8.2)	18	(8.0)	
Grading							
G1	220	(35.8)	138	(35.4)	82	(36.4)	*p* = 0.951
G2	314	(51.1)	201	(51.5)	113	(50.2)	
G3	81	(13.2)	51	(13.1)	30	(13.3)	
Pathological T stage							
pT1	122	(19.8)	78	(20.0)	44	(19.6)	*p* = 0.716
pT2	387	(62.9)	246	(63.1)	141	(62.7)	
pT3	106	(17.2)	66	(16.9)	40	(17.8)	
Pathological N stage							
pN0	491	(79.8)	311	(79.7)	180	(80.0)	*p* = 0.613
pN1	58	(9.4)	33	(8.5)	25	(11.1)	
pN2a	34	(5.5)	22	(5.6)	12	(5.3)	
pN2b	16	(2.6)	13	(3.3)	3	(1.3)	
pN2c	4	(0.7)	3	(0.8)	1	(0.4)	
pN3	12	(2.0)	8	(2.1)	4	(1.8)	
N upstaging							
No	491	(79.8)	311	(79.7)	180	(80.0)	*p* = 0.939
Yes	124	(20.2)	79	(20.3)	45	(20.0)	
Number of metastases							
1	66	(10.7)	34	(8.7)	32	(14.2)	*p* = 0.054
2	22	(3.6)	18	(4.6)	4	(1.8)	
3	24	(3.9)	18	(4.6)	6	(2.7)	
≥ 4	12	(2.0)	9	(2.3)	3	(1.3)	
Extranodal extension							
Negative	103	(16.8)	71	(17.4)	37	(15.6)	*p* = 0.462
Positive	16	(2.6)	8	(2.1)	8	(3.6)	
No metastasis	491	(80.7)	311	(80.5)	180	(80.9)	
Adjuvant treatment							
Radiotherapy	383	(62.3)	246	(63.1)	137	(60.9)	*p* = 0.590
Chemotherapy	232	(37.7)	144	(36.9)	88	(39.1)	

^a^ Mann–Whitney test.

Neck dissection was unilateral in 72.0% and bilateral in 28.0%, with homogeneous distributions of dissected levels on the left and right sides between groups (Table 2). In the BMF cohort, 61.3% received a facial artery flap (FAMM), 33.1% a tunnelized facial artery myomucosal island flap (t‐FAMMIF), and 5.6% a FAMMIF; in the FFF cohort, 93.8% received a RFFF, 5.3% an ALT flap, and 0.4% each a lateral arm or vastus lateralis flap (Table 2). No significant differences were found in terms of adjuvant radio/chemotherapy treatment (*p* = 0.590).

**TABLE 2:** Distribution of patients according to surgical data.

	All (*n* = 615)		Cases (*n* = 390)		Controls (*n* = 225)		*χ* ^2^ test
	*N*	(%)	*N*	(%)	*N*	(%)	
Neck dissection							
Unilateral	443	(72.0)	281	(72.1)	162	(72.0)	*p* = 0.989
Bilateral	172	(28.0)	109	(27.9)	63	(28.0)	
Type of flap							
FAMM			239	(61.3)	—		n.a.
t‐FAMMIF			129	(33.1)	—		
FAMMIF			22	(5.6)	—		
RFFF			—		211	(93.8)	
ALT			—		12	(5.3)	
Lateral arm			—		1	(0.4)	
Vastus lateralis			—		1	(0.4)	
Flap side							
Left	236	(36.6)	134	(34.4)	91	(40.4)	*p* = 0.131
Right	390	(63.4)	256	(65.6)	134	(59.6)	

**FIGURE 1:**
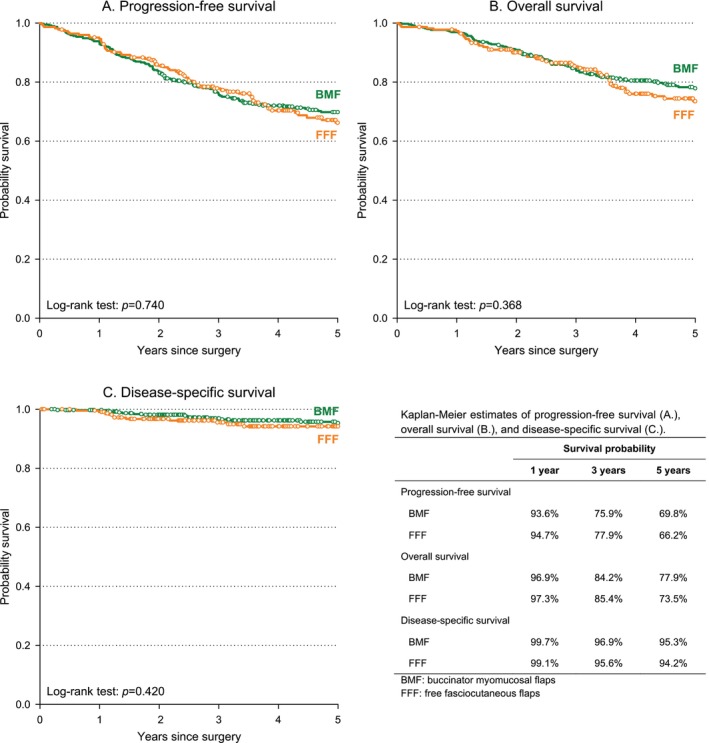
Progression‐free survival (A), overall survival (B), and disease‐specific survival (C) in patients undergoing reconstruction with buccinator myomucosal flap (BMF) or free fasciocutaneous flap (FFF). [Color figure can be viewed at wileyonlinelibrary.com]

Oncologic outcomes were comparable between reconstruction groups. Five‐year PFS (Figure 1A) was 69.8% (BMF) versus 66.2% (FFF), with HR 1.05 (95% CI: 0.78–1.43) univariable and 0.87 (95% CI: 0.43–1.78) multivariable (Table 3). Similarly, 5‐year OS (Figure 1B and Table 3) estimates were 77.9% (BMF) and 73.5% (FFF), with an HR for FFF versus BMF of 1.18 (95% CI: 0.82–1.68) univariable and 1.15 (95% CI: 0.48–2.76) multivariable. Five‐year DSS was also comparable, being 95.3% in BMF versus 94.2% in FFF (Figure 1C).

**TABLE 3:** Hazard ratio (HR), and corresponding 95% confidence interval (CI), of death, PFS‐event, local recurrence, regional recurrence, and distant recurrence for patients undergoing reconstruction with free fasciocutaneous flap (FFF) versus buccinator myomucosal flap (BMF).

Outcome	5‐year estimate		Univariate HR (95% CI)^a^	Multivariable HR (95% CI)^b^
	BMF	FFF		
Survival				
Progression‐free survival	69.8%	66.2%	1.05 (0.78–1.43)	0.87 (0.43–1.78)
Overall survival	77.9%	73.5%	1.18 (0.82–1.68)	1.15 (0.48–2.76)
Disease‐specific survival	95.3%	94.2%	1.38 (0.63–3.00)	0.42 (0.08–2.06)
Cumulative incidence				
Local recurrence^c^	7.5%	8.3%	1.04 (0.56–1.92)	0.75 (0.19–2.91)
Regional recurrence^c^	6.3%	6.1%	0.97 (0.49–1.90)	0.27 (0.06–1.19)
Distant recurrence^c^	2.1%	1.7%	0.69 (0.18–2.61)	1.24 (0.12–13.12)

^a^ Estimated from Cox proportional hazards model.

^b^ Adjusted for gender, age, cancer site, pathological T stage, pathological N stage upgrade, grading, extramodular extension, and adjuvant treatment.

^c^ Adjusted for competing risks according to the Fine–Gray model.

**FIGURE 2:**
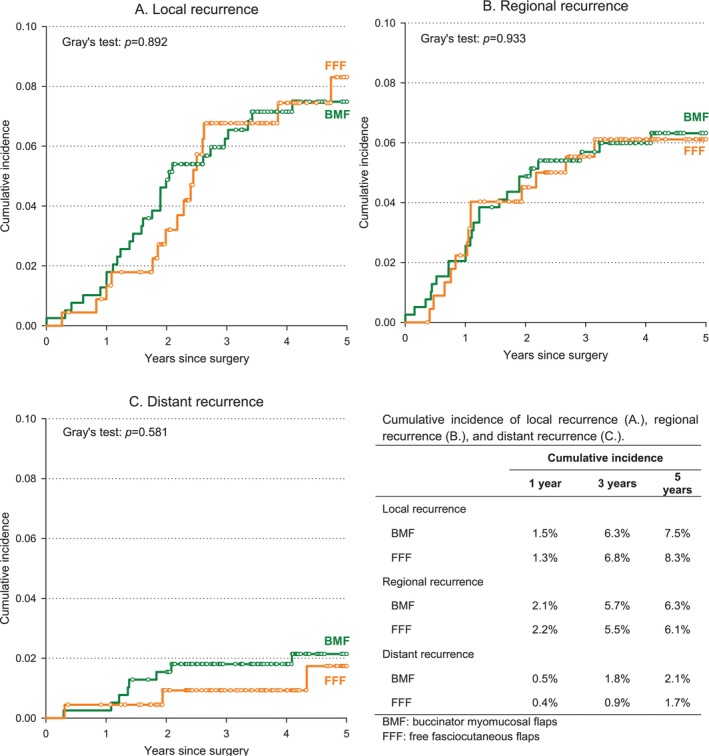
Cumulative incidence of local recurrence (A), regional recurrence (B), and distant recurrence (C) in patients undergoing reconstruction with buccinator myomucosal flap (BMF) or free fasciocutaneous flap (FFF). [Color figure can be viewed at wileyonlinelibrary.com]

In competing‐risk analyses, the 5‐year cumulative incidence of local recurrence was 7.5% (BMF) versus 8.3% (FFF) with adjusted HR 0.75 (95% CI: 0.19–2.91) (Figure 2A); regional recurrence 6.3% versus 6.1% with adjusted HR 0.27 (95% CI: 0.06–1.19) (Figure 2B); and distant recurrence 2.1% versus 1.7% with adjusted HR 1.24 (95% CI: 0.12–13.12) (Figure 2C). These results met the predefined non‐inferiority criterion for the primary endpoint (lower bound of the 95% CI for the adjusted HR < 1).

## 4 Discussion

In this multicenter case–control study of cT1–T3 cN0 SCC of the tongue and/or the oral floor, reconstruction with facial‐vessel–based BMF yielded oncologic outcomes comparable to those obtained with FFF. Five‐year OS and PFS did not differ significantly between groups, and the adjusted HR for PFS met the prespecified non‐inferiority criterion. Local, regional, and distant failure patterns were likewise similar. Collectively, these data indicate that preserving the facial artery and vein during selective neck dissection does not compromise oncologic safety when meticulous level I dissection is performed in appropriately selected cN0 patients.

Our results extend and strengthen the only prior series specifically assessing oncologic safety of facial‐vessel preservation in BMF reconstruction. In that single‐center cohort, Ferrari et al. [25] reported no increase in regional recurrences after preservation of the facial artery/vein during elective neck dissection, but the study was limited by small size, site/stage heterogeneity, and lack of a control group reconstructed with free flaps. By contrast, the present study is multicenter, focuses on a homogeneous disease compartment (tongue/oral floor cN0), and includes a control group in which the facial vessels were sacrificed.

The oncologic acceptability of preserving facial vessels has been more extensively studied with the submental island flap, where comparative series suggest no detriment in disease control when careful perivascular nodal clearance is performed [22, 23, 24]. This aligns with surgical pathology data emphasizing that perifacial nodes in level I can harbor disease and thus warrant meticulous clearance during selective neck dissection [29].

Our cohort's 20.2% nodal upstaging from cN0 to pathologic N+ is consistent with contemporary estimates of occult metastasis in early oral tongue and oral floor cancer [30, 31]. Despite this, in our series, whether facial vessels were sacrificed or preserved was uninfluential on regional recurrence, which remained comparable between groups. It is worth noting that among patients who were upstaged, a small proportion presented with pN2a disease despite being clinically N0. These cases likely represented single ipsilateral lymph node metastases measuring between 3 and 6 cm located deep in the submandibular or perifacial compartments, where limited external contour changes can make detection difficult on physical examination and imaging. This phenomenon has been previously described in cN0 oral cavity cancers, in which the accuracy of clinical and radiologic neck assessment remains limited [32, 33]. Similarly, all pN3 cases in our series corresponded to pN3b disease with extranodal extension, while no nodal metastases > 6 cm (pN3a) were observed.

A frequently voiced theoretical concern is that using intraoral tissues exposed to the same carcinogenic field might predispose to metachronous malignancies in the flap. The underlying concept of field cancerization is well established in head and neck oncology, from the classic description by Slaughter et al. [34] to modern genetic models by Braakhuis et al. [35]. While the absolute risk of second primaries arising on reconstruction flaps is low, case reports and systematic reviews document such events, predominantly on cutaneous paddles after intraoral transfer, sometimes decades later, reinforcing the need for long‐term surveillance irrespective of the reconstructive method [36, 37, 38]. Importantly, our study was not designed to quantify this rare outcome; nevertheless, the similar local control and regional failure rates between BMF and FFF groups argue against any early increased oncologic hazard attributable to facial‐vessel preservation or to the use of intraoral mucosa for reconstruction.

For cN0 T1–T3 tongue/oral floor cancers managed with selective neck dissection, our findings support BMF reconstruction as an oncological non‐inferior alternative to FFF when defects are small to medium and a pull‐through resection is not required, level I is dissected with meticulous perifacial nodal clearance, and surgeon expertise with facial‐vessel–based harvest is available. This approach leverages mucosal like‐with‐like replacement [9, 10, 11, 12, 13, 14, 15, 16, 39] and potential sensory recovery [17, 40], while avoiding extraoral donor morbidity in suitable cases. In patients with cN+ necks, especially with level IB metastases, the use of facial‐vessel–pedicled BMFs should be considered with great caution. Perifacial nodes can harbor a substantial burden of disease, including occult metastases, and oncologic safety hinges on uncompromised clearance of this compartment [29, 41]. Also, the surgeon needs a back‐up plan as he might need to sacrifice the facial vessels if the node metastasis is adherent to them. In such scenarios, pedicles that do not rely on the facial vessels may be preferable: a buccal‐artery myomucosal flap can be harvested while sacrificing the facial vessels, using a pedicle based on the buccal branch of the internal maxillary artery [9, 19, 42]. Alternatively, when lymph node metastases are unilateral, a contralateral t‐FAMMIF can be considered [43, 44, 45]; tunnelization through the neck substantially enlarges the arc of rotation, enabling reconstruction of contralateral sites without compromising oncologic principles [46].

This study has some limitations that must be acknowledged. First, its retrospective, multicenter nature over nearly a decade introduces potential selection and performance biases, as well as practice heterogeneity (including surgeon preference and institutional pathways). Second, the control cohort reconstructed with FFF (*n* = 225) was smaller than the per‐group sample size projected by our non‐inferiority calculation (379 per group), which may have reduced statistical power and widened CIs, especially for secondary endpoints and multivariable and competing‐risk estimates. However, post hoc power analysis confirmed that the non‐inferiority analysis of PFS had a power > 90%. Third, allocation to reconstruction type was nonrandomized and based on clinical judgment, creating the possibility of indication bias that may not be fully mitigated by adjustment. Fourth, although most recurrences occur within the first 24 months, a minimum follow‐up of 2 years may still be insufficient to detect late regional or distant metastases. Nevertheless, the mean follow‐up in our series exceeded four and a half years, providing a robust observation period for most patients. Fifth, central pathology review and independent adjudication of recurrence were not performed; consequently, minor misclassification of pathologic variables and failure sites, as well as variability in surveillance protocols, may have occurred. Residual confounding from unmeasured factors is therefore possible. Nevertheless, strengths include the largest series to date focused on cN0 tongue/oral floor SCC, disease‐site and stage standardization (T1–T3; cN0), an explicit control group in which facial vessels were sacrificed, and application of risk‐adjusted survival and competing‐risk methods.

## 5 Conclusions

Facial‐vessel–based BMFs achieved oncologic outcomes comparable to FFF, meeting the prespecified non‐inferiority criterion for PFS and showing similar local, regional, and distant failure patterns. These data indicate that, with meticulous level I clearance, preservation of the facial artery and vein does not compromise oncologic safety, and BMFs are a valid option for small‐to‐medium defects of the tongue and/or oral floor in cN0 patients when a pull‐through resection is not required.

## Conflicts of Interest

The authors declare no conflicts of interest.

## Data Availability

The data that support the findings of this study are available from the corresponding author upon reasonable request.
